# Cilia-Associated Genes Play Differing Roles in Aminoglycoside-Induced Hair Cell Death in Zebrafish

**DOI:** 10.1534/g3.116.030080

**Published:** 2016-05-19

**Authors:** Tamara M. Stawicki, Liana Hernandez, Robert Esterberg, Tor Linbo, Kelly N. Owens, Arish N. Shah, Nihal Thapa, Brock Roberts, Cecilia B. Moens, Edwin W. Rubel, David W. Raible

**Affiliations:** * Department of Biological Structure, University of Washington, Seattle, Washington 98195; † Virginia Merrill Bloedel Hearing Research Center, University of Washington, Seattle, Washington 98195; ‡ Department of Otolaryngology, Head and Neck Surgery, University of Washington, Seattle, Washington, 98195; § Division of Basic Sciences, Fred Hutchinson Cancer Research Center, Seattle, Washington, 98109

**Keywords:** aminoglycosides, cilia, hair cells, intraflagellar transport, transition zone

## Abstract

Hair cells possess a single primary cilium, called the kinocilium, early in development. While the kinocilium is lost in auditory hair cells of most species it is maintained in vestibular hair cells. It has generally been believed that the primary role of the kinocilium and cilia-associated genes in hair cells is in the establishment of the polarity of actin-based stereocilia, the hair cell mechanotransduction apparatus. Through genetic screening and testing of candidate genes in zebrafish *(Danio rerio)* we have found that mutations in multiple cilia genes implicated in intraflagellar transport (*dync2h1*, *wdr35*, *ift88*, and *traf3ip)*, and the ciliary transition zone (*cc2d2a*, *mks1*, and *cep290*) lead to resistance to aminoglycoside-induced hair cell death. These genes appear to have differing roles in hair cells, as mutations in intraflagellar transport genes, but not transition zone genes, lead to defects in kinocilia formation and processes dependent upon hair cell mechanotransduction activity. These mutants highlight a novel role of cilia-associated genes in hair cells, and provide powerful tools for further study.

Cilia are microtubule-based projections located on the apical surface of cells. One class of cilia found on the majority of cells in multicellular organisms, called primary cilia, are nonmotile and important for cellular signaling (Satir and Christensen 2007; Berbari *et al.* 2009). Cilia are formed and maintained through regulated microtubule-dependent protein trafficking along their length, a process known as intraflagellar transport (IFT). Anterograde IFT, transport from the cell body to the ciliary tip, depends on kinesin-2 motors and the IFT-B complex of adaptor proteins. Retrograde IFT, transport from the tip back to the base, depends on dynein-2 motors and the IFT-A complex of adaptor proteins (Scholey 2003; Pedersen *et al.* 2006). The entry and exit of protein into the cilia is further controlled by a group of proteins localized at the cilia base, in a region known as the transition zone, that act as a molecular gate (Reiter *et al.* 2012; Blacque and Sanders 2014).

Mutations in cilia-associated genes lead to a broad spectrum of diseases known as ciliopathies. These diseases affect a range of organ systems with different symptoms associated with each disease. In some cases, a single gene is implicated in multiple ciliopathies (Badano *et al.* 2006; Waters and Beales 2011). Mutations in IFT genes frequently lead to skeletal ciliopathies characterized by a shortening of bones and, in some cases, polydactyly (Huber and Cormier-Daire 2012). Mutations in transition zone genes are frequently seen in ciliopathies associated with renal disease and/or retinal degeneration (Czarnecki and Shah 2012). In a subset of cases, including those resulting in Alström Syndrome, Bardet-Biedl Syndrome, Usher Syndrome, and autosomal recessive deafness DFNB66, mutations in cilia-associated genes have been shown to cause hearing loss (Ross *et al.* 2005; Adams *et al.* 2007; Grati *et al.* 2015).

Hair cells, the sensory cells of the auditory and vestibular systems, contain a single primary cilium known as the kinocilium. Auditory hair cells of many species lose their kinocilia during development whereas kinocilia are maintained in vestibular hair cells (Tanaka and Smith 1978; Lim and Anniko 1985; Ernstson and Smith 1986). The apical surfaces of hair cells also contain actin-based stereocilia that develop in rows, in order of ascending height. The stereocilia gate ions flow from the surrounding medium in response to mechanical stimuli, thus activating hair cells through a process known as mechanotransduction (Schwander *et al.* 2010). The kinocilium is adjacent to the tallest stereocilia row. While kinocilia may play a role in hair cell activity at early stages (Kindt *et al.* 2012), mechanotransduction activity in mature hair cells is entirely due to the stereocilia (Hudspeth and Jacobs 1979). It is generally believed that the primary role of the kinocilium and cilia-associated genes in auditory hair cells is in the establishment of stereocilia polarity (Ross *et al.* 2005; Jones *et al.* 2008), a process also known to be dependent upon the planar cell polarity pathway (Montcouquiol *et al.* 2003; Wang *et al.* 2005, 2006).

However, there are reasons to believe that cilia-associated genes may be playing additional roles in hair cells. In mice deficient in the ciliary basal body gene responsible for Alström syndrome, *Alms1*, defects in stereocilia morphology are present early in development, however, hearing loss shows a delayed onset. This suggests the stereocilia morphology defects are not solely responsible for hearing loss (Jagger *et al.* 2011). Recent evidence has shown that genes that are traditionally thought of as cilia genes can also have cellular functions independent of the cilium. IFT genes have been implicated in protein trafficking and cytoskeletal organization in nonciliated cells (Finetti *et al.* 2009; Delaval *et al.* 2011; Cong *et al.* 2014), and mutations in cilia-associated genes have been shown to lead to increased DNA damage (Choi *et al.* 2013; Slaats *et al.* 2015). This leaves open possible roles for cilia-associated genes in hair cells after the developmental loss of the kinocilium. Indeed, a number of cilia-associated genes remain expressed in mammalian auditory hair cells after kinocilia loss (Liu *et al.* 2008).

It is well known that aminoglycoside antibiotics cause hearing loss and vestibular dysfunction in human patients (Moore *et al.* 1984; Lerner *et al.* 1986). Through genetic screening for modulators of sensitivity to aminoglycoside exposure, using the zebrafish lateral line system, we have discovered that mutations in multiple cilia-associated genes lead to resistance to aminoglycoside-induced hair cell death. We have previously reported that a mutation in the cilia transition zone gene *cc2d2a* leads to resistance to the aminoglycoside neomycin (Owens *et al.* 2008). Here, we report that mutations in the cilia transition zone gene *mks1*, as well as the basal body/transition zone gene *cep290*, also lead to moderate protection against neomycin-induced hair cell death. Additionally, mutations in the IFT genes *dync2h1*, *wdr35*, *ift88*, and *traf3ip* strongly protect against neomycin-induced hair cell death. Mutations in these genes appear to differently affect hair cells, as IFT but not transition zone mutants show defects in kinocilia formation and aminoglycoside uptake. As stereocilia morphology appears grossly normal in these mutants, we believe that this work reveals a novel role for cilia-associated genes in hair cells. It also suggests that antibiotic resistance may be a useful phenotype for discovering other cilia-related genes that play a role in mature hair cells, while working toward uncovering the full role of cilia-associated genes in regulating hair cell structure and function.

## Materials and Methods

### Animals

All experiments used 5 d post-fertilization (dpf) *Danio rerio* (zebrafish) larvae, unless otherwise noted. Mutant alleles were maintained as heterozygotes in the *AB wild-type strain, and experiments were carried out in this strain background. For *arl13b^hi459Tg^*, *cc2d2a^w38^*, *cc2d2a^w123^*, *dync2h1^w46^*, *ift88^tz288^*, and *traf3ip^tp49d^*, mutants were separated from wild-type siblings based on associated morphological phenotypes. Both *ccd2d2a* and *dync2h1* mutants can be consistently identified by their sinusoidal body shape (Owens *et al.* 2008; Ryan *et al.* 2013), whereas *arl13b*, *ift88*, and *traf3ip* mutants had a c-shaped body (Tsujikawa and Malicki 2004; Omori *et al.* 2008; Duldulao *et al.* 2009). For *mks1^w152^* and *wdr35^w150^*, genotyping was used to distinguish between mutants and wild-type siblings. For the *cep290^fh378^* mutant strain, genotyping was always performed to confirm animals as wild-type siblings or mutants; however, in some experiments, animals were presorted based on body morphology. Genetic mapping used the Wik strain. Larvae were raised in embryo media (EM) consisting of 1 mM MgSO_4_, 150 μM KH_2_PO_4_, 42 μM Na_2_HPO_4_, 1 mM CaCl_2_, 500 μM KCl, 15 mM NaCl, and 714 μM NaHCO_3._ The University of Washington Institution Animal Care and Use Committee approved all experiments. Mutant strains used in this study are available upon request.

### Genetic screening

F2 mutant families were generated as previously described (Owens *et al.* 2008). To screen F2 families, adult pairs were incrossed and 15–50 offspring per pair were treated with 200 μM neomycin at 5 dpf. Fish were screened for neomycin resistance using the vital dye DASPEI [2-(4-dimethylaminostyryl)-N-ethylpyridinium iodide] (Molecular Probes), and the numbers of animals per clutch showing either a typical or atypical neomycin response were counted. Pairs where approximately one quarter of all animals showed an atypical response were rescreened to confirm the phenotype and then outcrossed with *AB wild-type fish. Approximately 500 F2 families were screened with at least 4–6 pairs screened per family when possible.

### Genetic mapping

The molecular bases of mutations first found by phenotypic screening were identified by genetic mapping using the polymorphic Wik strain. Hybrid *AB/Wik mutant carriers were intercrossed to generate progeny for linkage marker analysis. Mutant and wild-type fish were selected based on susceptibility to 200 μM neomycin. Microsatellite markers for each chromosome (Knapik *et al.* 1998; Shimoda *et al.* 1999) were amplified by PCR and tested for cosegregation with mutant phenotypes. Pools of 20 mutants and 20 wild-type siblings were used for bulk segregant analysis. Markers cosegregating with the mutant allele were further evaluated with individual DNA from 213 mutants and 16 wild-type siblings for the *dync2h^w46^* mutation, and 508 mutants and 73 wild-type siblings for the *wdr35^w150^* mutation. In addition to the existing microsatellite markers, candidate simple sequence repeat (SSR) marker primer pairs were generated using the Zebrafish Genome SSR search website (Massachusetts General Hospital, Charlestown MA 02129; World Wide Web URL: http://danio.mgh.harvard.edu/chrMarkers/zfssr.html). To sequence candidate genes following linkage mapping, RNA was isolated from pools of 20 wild-type sibling or mutant embryos using TRIzol Reagent (Ambion), and cDNA was prepared using SuperScript III Reverse Transcriptase (Invitrogen). Genes were amplified by PCR from the resultant cDNA and then sent to Eurofins MWG Operon for sequencing.

### CRISPR and TALEN mutagenesis

To generate *mks1* mutants, guide RNA (gRNA) was designed to two different target sites: 5′-GGAGGCCGTCTGAGTGCTGA-3′ and 5′-GTGTGATACTGACGCTCCAG-3′. Targets were selected using the design tool at http://crispr.mit.edu. Cas9 mRNA and gRNA were synthesized as previously described (Shah *et al.* 2015). Embryos were injected with 1 nl of a solution containing 200 ng/μl of Cas9 mRNA and 50 ng/μl each of the two gRNAs. Transmission of a genetic change at the gRNA target site was screened for by performing a PCR using primers flanking the target and running the product on a 3% lithium borate gel (Brody *et al.* 2004) to look for size changes. Experiments were performed on larvae in the F3 generation containing a 25 bp insertion at the first target site (Supplemental Material, Table S1).

To generate *cep290* mutants, a pair of TALENs was designed for exon 6 of the *cep290* coding sequence. The first TALE binds the sequence 5′-CTGCCTCACTTCCTGTCCA-3′ and the second binds the sequence 5′-TTGTCTCCCCCTCCCATCA-3′. TALENS were generated as previously described (Sanjana *et al.* 2012). TALEN mRNA was generated and polyadenylated using the T7 ULTRA Transcription Kit (Ambion), and RNA was purified using a standard phenol-chloroform extraction. Zebrafish embryos were injected at the one-cell stage with 200 pg of each TALEN mRNA. F0 founders of *fh378* were identified and F1 heterozygotes were raised to establish the mutant strain.

### Neomycin treatment

Fish were treated with the designated concentration of neomycin (Sigma-Aldrich) dissolved in EM for 30 min at 28.5°. They were then washed three times in EM, and left to recover for 1 hr. At the end of recovery, animals used for hair cell counts were fixed for immunostaining. Hair cells were counted in the OP1, M2, IO4, O2, MI2, and MI1 neuromasts (Raible and Kruse 2000). All hair cell number and neomycin response data were obtained through hair cell counts. For hair cell data presented as the percentage of controls, the average number of hair cells/neuromast was calculated for each individual fish by dividing the total number of hair cells counted per fish by the number of neuromasts analyzed. This number was then divided by the average number of hair cells/neuromast for control animals, and expressed as a percentage. Fish that were not treated with neomycin were used as controls. Animals used for genetic mapping were screened for neomycin resistance using the vital dye DASPEI (Molecular Probes). They were exposed to DASPEI at a final concentration of 0.05% for 15 min. Neuromasts SO1, SO2, IO1, IO2, IO3, IO4, O2, M2, MI1, and MI2 were scored as previously described (Harris *et al.* 2003). Animals with a score of eight or higher were considered resistant.

### Immunohistochemistry

Fish used for immunohistochemistry were fixed for 2 hr at room temperature in 4% paraformaldehyde. Antibody labeling was carried out as previously described (Stawicki *et al.* 2014). Fish used for hair cell counts were labeled with a mouse anti-parvalbumin primary antibody (Millipore, MAB1572) diluted at 1:500 in antibody block (5% heat-inactivated goat serum in PBS, 0.2% Triton, 1% DMSO, and 0.2% BSA). Fish used for the analysis of cilia morphology were labeled with a mouse anti-acetylated tubulin primary antibody (Sigma-Aldrich, 6-11B-1) diluted at 1:1000 in antibody block. For phalloidin labeling, after fixation fish were incubated for 2 hr at room temperature in Alexa Fluor 488 Phalloidin (Molecular Probes) diluted 1:100 in antibody block. They were then washed two times in PBST, one time in PBS, and stored in a 50:50 PBS and glycerol mixture prior to use.

### Uptake experiments

Fish were treated with either 2.25 μM FM1-43 FX (Molecular Probes) for 1 min, or 25 μM neomycin-Texas Red (TR) for 5 min (neomycin-TR was made as previously described; Stawicki *et al.* 2014). They were then washed three times in EM and anesthetized with MS222 for imaging. Images were obtained and analyzed using SlideBook software on a Marianas spinning disk confocal system (Intelligent Imaging Innovations). For each animal, a single neuromast was imaged and analyzed. A stack of 30 1-μm optical sections was obtained and maximum projection images were analyzed. The average fluorescence intensity of the cell bodies of the neuromast was calculated and divided by the background fluorescence of the image. The fluorescence measurements of mutant neuromasts were then normalized to the average fluorescence intensity of wild-type sibling controls imaged on the same day with the same imaging parameters.

### Data availability

The authors state that all data necessary for confirming the conclusions presented in the article are represented fully within the article.

## Results

### Mutations in cilia-associated genes lead to resistance to neomycin-induced hair cell death

**Figure 1:**
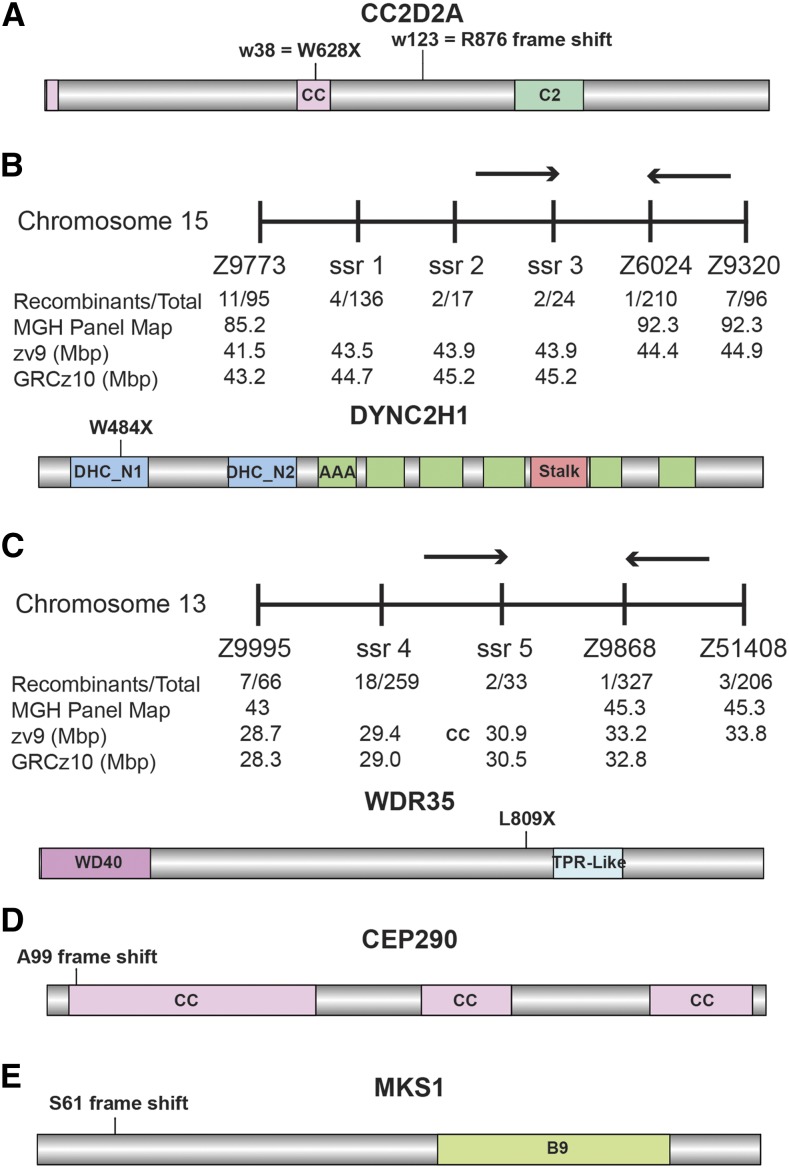
Identification of mutations in cilia-associated genes that confer resistance to aminoglycoside-induced hair cell death. (A) Two mutations have been identified in the *cc2d2a* gene. One mutation (*w38*) (W628X; Owens *et al.* 2008) causes a premature stop codon in a coiled-coil (CC) domain. The other (*w123*) leads to the retention of the intron between exons 20 and 21 causing a frameshift. Both pink boxes are putative CC domains. (B) Top: neomycin-resistant mutant allele *w46* mapped to a region of approximately 0.5 Mb on chromosome 15. The microsatellite markers used for mapping are shown, as well as the number of recombinant animals at each position. Bottom: sequencing of the *dync2h1* gene contained in this region identified a G to A nucleic acid change in mutants at residue 1452, leading to a premature stop codon in the N-terminus of the protein (W484X). All green boxes represent AAA domains (ATPases Associated with diverse cellular Activities). (C) Top: neomycin-resistant mutant allele *w150* mapped to a region of approximately 2.3 Mb on chromosome 13. The microsatellite markers used for mapping are shown, as well as the number of recombinant animals at each position. Bottom: sequencing of the *wdr35* gene contained in this region identified a T to A nucleic acid change in mutants at residue 2426, leading to a premature stop codon just upstream of the tetratricopeptide repeat TPR-Like domain (L809X). (D and E) Frameshift mutations were generated in the N-terminus of both Cep290 (D) and Mks1 (E) using genome editing techniques. The *cep290* mutation was a 2 bp deletion, resulting in a frameshift and truncated coding sequence after A99, whereas the *mks1* mutation was a 25 bp insertion causing a frameshift and truncated coding sequence after S61. Due to the large range of sizes, the individual protein images are not to the same scale. DHC, dynein heavy chain domain.

To identify novel genes involved in aminoglycoside-induced hair cell death we performed a genetic screen looking for mutants resistant to the effects of exposure to neomycin. Through this screen we identified two mutants, *w46* and *w123*, that showed a sinusoidal body pattern similar to that previously seen in the *sentinel* mutant (*cc2d2a^w38^*; Owens *et al.* 2008). Through complementation testing we found that the *w123* allele, but not the *w46* allele, failed to complement *cc2d2a^w38^*. Sequencing showed that the intron between exons 20 and 21 is retained in *cc2d2a* cDNA isolated from the *w123* mutant, leading to a frame shift in the protein (Figure 1A and Table S1).

Genetic mapping of the *w46* allele localized the mutation to chromosome 15 (Figure 1B). The body morphology of *w46* mutants is similar to a previously reported *dync2h1* mutant (Ryan *et al.* 2013), one of the genes in the mapped interval. Dync2h1 is believed to be the motor protein responsible for retrograde IFT, the trafficking of protein from the tip to the base of cilia (Pazour *et al.* 1999; May *et al.* 2005). Sequencing of *dync2h1* cDNA in *w46* mutants identified a premature stop codon in the 5' region of the gene (Figure 1B and Table S1).

An additional mutant isolated in this screen, *w150*, had a grossly normal body morphology; genetic mapping localized the mutation to a region on Chromosome 13 containing the retrograde IFT gene *wdr35* (Figure 1C). Wdr-35 is part of the IFT-A complex, a group of proteins believed to serve as adaptors between the cargo and motors in retrograde IFT (Cole 2003; Mukhopadhyay *et al.* 2010). Sequencing of *wdr35* cDNA in the *w150* mutant strain identified a premature stop codon (Figure 1C and Table S1).

As three mutations identified in our screen are all implicated in cilia function, we tested additional mutations in cilia-associated genes for resistance to neomycin. We obtained existing mutations in the cilia gene *arl13b* (Golling *et al.* 2002), and anterograde IFT genes *ift88* (Tsujikawa and Malicki 2004) and *traf3ip* (Omori *et al.* 2008). Arl13b localizes to the cilia axonemal region and is believed to play roles in cilia formation and Sonic hedgehog signaling (Sun *et al.* 2004; Caspary *et al.* 2007; Duldulao *et al.* 2009; Larkins *et al.* 2011). Ift88 and Traf3ip are components of the IFT-B complex, the protein adaptors for anterograde IFT, and are also required for cilia formation (Pazour *et al.* 2000; Cole 2003; Kunitomo and Iino 2008; Berbari *et al.* 2011). Additionally, we used genome editing techniques to generate new mutations in the transition zone genes *mks1* and *cep290 (*Figure 1, D and E and Table S1). Mks1 and Cc2d2a are both part of a large complex of proteins implicated in human ciliopathies localized to the transition zone of cilia (Garcia-Gonzalo *et al.* 2011; Chih *et al.* 2012). Cep290 has also been shown to interact with Cc2d2a (Gorden *et al.* 2008; Garcia-Gonzalo *et al.* 2011), and is believed to bridge the basal body and transition zone (Yang *et al.* 2015).

**Figure 2:**
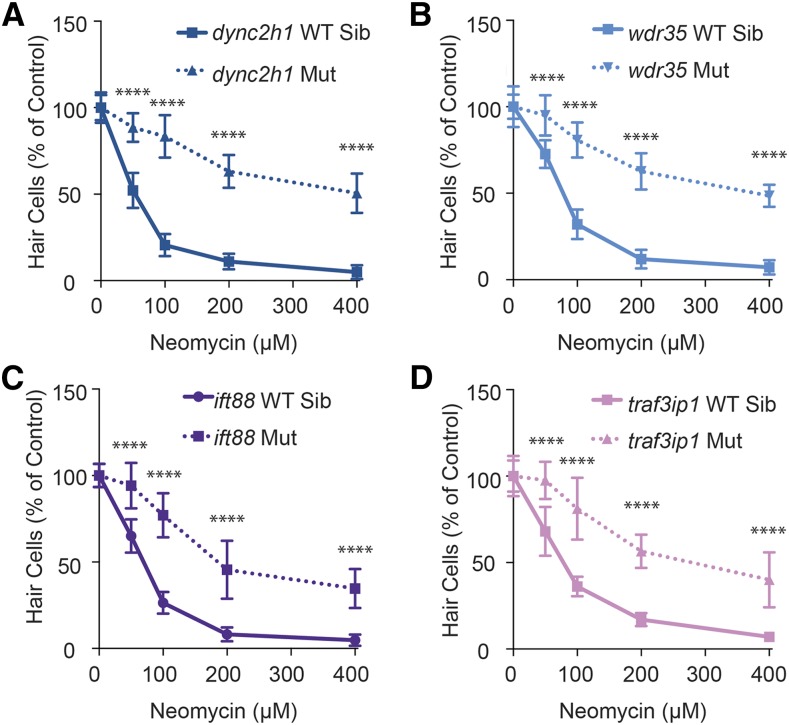
Mutations in intraflagellar transport (IFT) genes lead to resistance to neomycin-induced hair cell death across a range of neomycin doses. Mutants of the retrograde IFT genes *dync2h1* (A) and *wdr35* (B), as well as the anterograde IFT genes *ift88* (C) and *traf3ip* (D), show strong resistance to neomycin-induced hair cell death across a range of doses. Dose response curves for both wild-type siblings and mutants were independently normalized to either the wild-type sibling or mutant group of control fish not treated with neomycin. Data are displayed as mean ± SD. *n* = 10 fish for *dync2h1*, *ift88*, and *traf3ip*. *n* = 10–20 for *wdr35*. **** *P* < 0.00001 by two-way ANOVA and Šídák multiple comparisons test. Mut, mutant; WT Sib, wild-type sibling.

**Figure 3:**
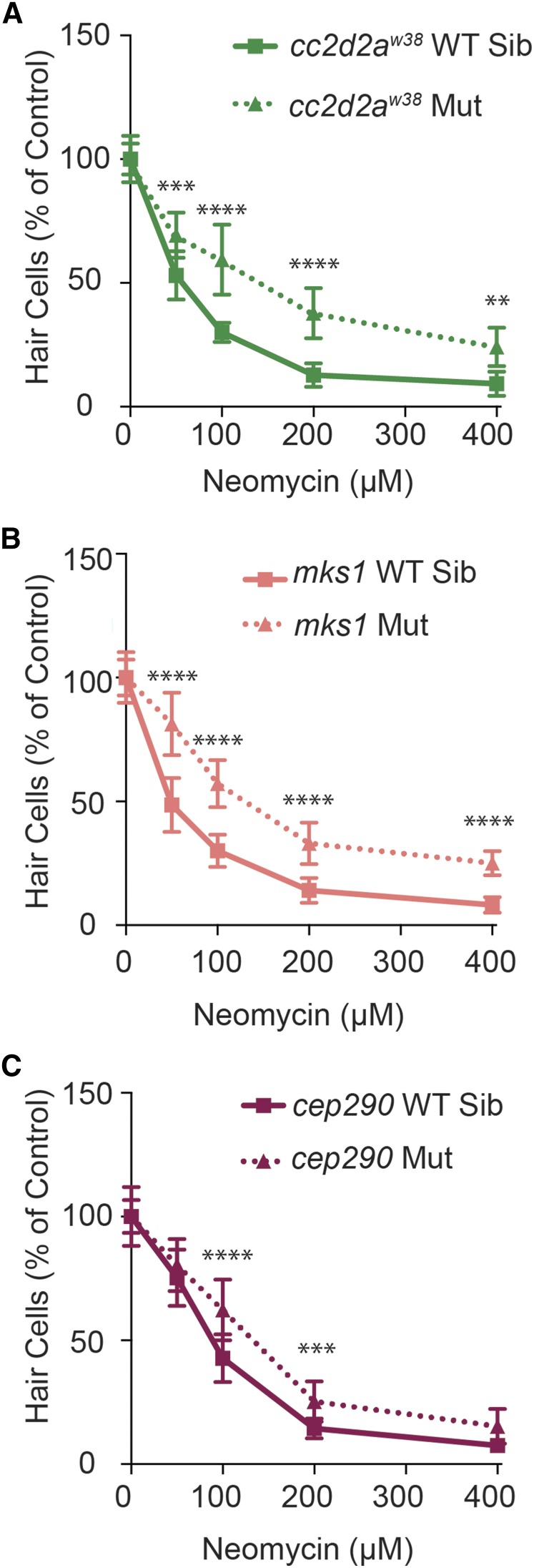
Mutations in transition zone genes lead to resistance to neomycin-induced hair cell death across a range of neomycin doses. Mutants of the transition zone genes *cc2d2a* (A), *mks1* (B), and *cep290* (C) show moderate resistance to neomycin-induced hair cell death. Dose response curves for both wild-type siblings and mutants were independently normalized to their no neomycin control hair cell numbers. Data are displayed as mean ± SD. *n* = 10 fish for *cc2d2a*, *n* = 11–22 for *cep290*, and *n* = 8–14 for *mks1*. ** *P* < 0.01, *** *P* < 0.001, and **** *P* < 0.00001 by two-way ANOVA and Šídák multiple comparisons test. Mut, mutant; WT Sib, wild-type sibling.

We found that all IFT and transition zone genes tested showed resistance to neomycin-induced hair cell death. In control animals, approximately 9–14% of hair cells remained in each neuromast after treatment with 200 μM neomycin. In IFT mutants, these numbers were increased to 48–70% (Table 1), with significant protection seen across the range of neomycin doses from 50–400 μM (Figure 2). Transition zone mutants showed slightly less resistance to neomycin-induced hair cell death than IFT mutants. *cc2d2a* and *mks1* mutants had 24–30% hair cells remaining following treatment with 200 μM neomycin, and *cep290* mutants had 18% remaining (Table 1). *cc2d2a* and *mks1* mutants additionally showed significant protection across a range of neomycin doses from 50–400 μM, whereas protection in *cep290* mutants was limited to 100 and 200 μM neomycin (Figure 3). *arl13b* mutants failed to show any protection against neomycin-induced hair cell death (Table 1). IFT mutants, but not transition zone mutants, additionally showed a decrease in control hair cell numbers as compared to their wild-type siblings (Table 1). Decreases in hair cell number have previously been shown in the inner ear of *ift88* zebrafish mutants (Tsujikawa and Malicki 2004).

**Table 1:** Mutations in numerous cilia genes leads to resistance in neomycin-induced hair cell death

	Wild-Type Sibling			Mutant			
Gene	Control	200 μM Neomycin	% HCs Remaining	Control		200 μM Neomycin	% HCs Remaining
Ciliary axoneme and intraflagellar transport							
* dync2h1 (w46)*	12.13 ± 1.15	1.05 ± 0.33	8.65	8.65 ± 1.27		5.63 ± 1.26	65.13
				*P* < 0.0001		*P* < 0.0001	
* wdr35 (w150)*	11.37 ± 0.92	1.84 ± 0.63	16.18	10.10 ± 0.89		6.17 ± 1.41	61.89
				*P* = 0.0002		*P* < 0.0001	
* ift88 (tz288)*	12.38 ± 1.78	1.17 ± 0.71	9.42	9.27 ± 0.65		4.43 ± 1.70	47.74
				*P* < 0.0001		*P* < 0.0001	
* traf3ip1 (tp49d)*	11.6 ± 1.43	1.08 ± 0.55	9.34	6.73 ± 0.79		4.78 ± 0.69	71.04
				*P* < 0.0001		*P* < 0.0001	
* arl13b (hi459Tg)*	11.78 ± 1.54	1.65 ± 0.85	14.00	11.92 ± 1.01		1.30 ± 0.37	10.88
				*P* = 0.9556		*P* = 0.7145	
Transition zone							
* cc2d2a (w38)*	11.10 ± 1.72	1.53 ± 0.69	13.78	12.33 ± 1.93		3.68 ± 0.47	29.85
				*P* = 0.0975		*P* = 0.0023	
* cc2d2a (w123)*	12.78 ± 2.5	1.17 ± 0.38	9.13	12.73 ± 2.24		3.05 ± 0.48	23.95
				*P* = 0.9973		*P* = 0.0373	
* mks1 (w152)*	12.34 ± 0.86	1.26 ± 0.46	10.21	12.17 ± 0.83		2.88 ± 0.65	23.66
				*P* = 0.6768		*P* < 0.0001	
* cep290 (fh378)*	12.30 ± 1.02	1.44 ± 0.34	11.70	12.19 ± 1.04		2.24 ± 0.88	18.34
				*P* = 0.7217		*P* = 0.0244	

Numbers are average number of hair cells/neuromast ± SD. Significance was calculated using an ANOVA (analysis of variance) and Šídák multiple comparisons test. *n* = 10 for *dync2h1*, *ift88*, *traf3ip*, *arl13b*, *cc2d2a^w38^*, and *cc2d2a^w123^*, *n* = 16 for *cep290*, *n* = 31–37 for wild-type siblings, and *n* = 8–14 for mutants of *wdr35* and *mks1*. HCs, hair cells.

**Figure 4:**
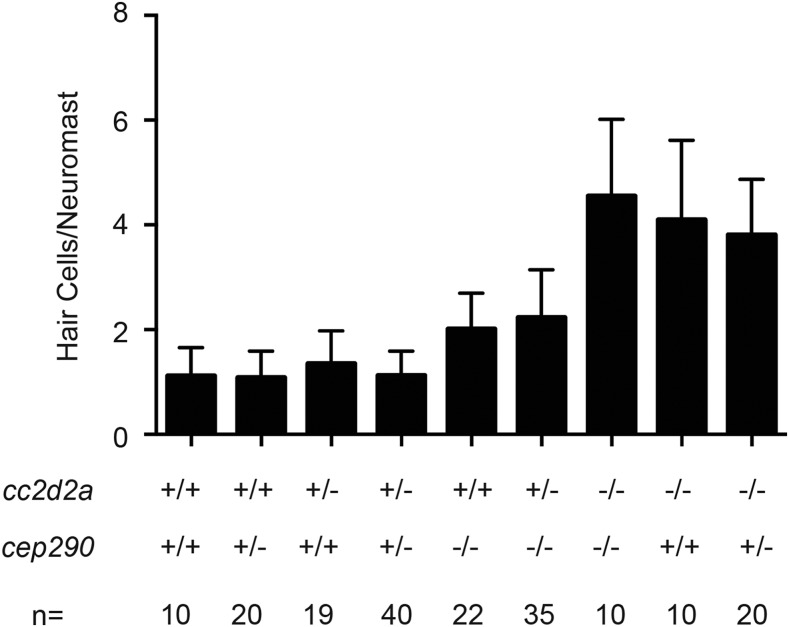
*cc2d2a* and *cep290* mutations do not show synergistic interactions. Hair cells per neuromast following 200 μM neomycin in animals with various combinations of *cc2d2a* and *cep290* mutant alleles. There does not appear to be any genetic interaction between the two mutant alleles. + is a wild-type allele, and − is a mutant allele. *n* = number of fish for each group. Data are displayed as mean + SD.

It has previously been reported that there was a synergistic genetic interaction when *cc2d2a^w38^* mutants were injected with *cep290* morpholino oligonucleotides (Gorden *et al.* 2008). To test if similar synergism was seen in terms of resistance to neomycin-induced hair cell death, potentially resulting in a level of resistance like that seen in IFT mutants, we generated *cc2d2a*; *cep290* double mutants. We found that the presence of either one or two copies of the other gene’s mutant allele had no additional synergistic effect on the amount of neomycin resistance seen in *cc2d2a* or *cep290* homozygous mutants (Figure 4), or on the number of hair cells under control conditions (data not shown).

### Kinocilia morphology is disrupted in IFT but not transition zone mutants

**Figure 5:**
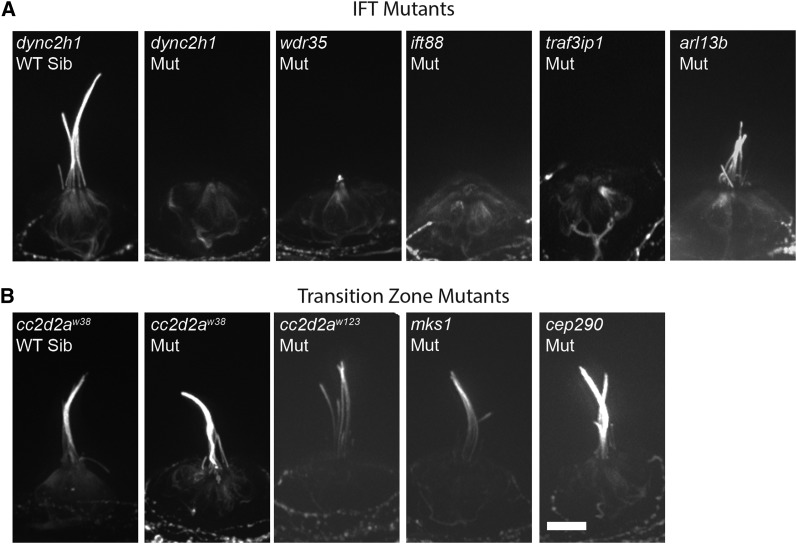
Intraflagellar transport (IFT) but not transition zone mutants show defects in kinocilia morphology. Neuromasts of 5 dpf zebrafish larvae stained with acetylated tubulin to label kinocilia. (A) Mutations in the IFT genes *dync2h1*, *wdr35*, *ift88*, and *traf3ip* cause a lack of kinocilia in cells. A mutation in the ciliary axoneme gene *arl13b* causes a shortening, but not an absence, of kinocilia. (B) Kinocilia morphology appears unchanged by mutations in the transition zone genes *cc2d2a*, *mks1*, and *cep290*. Images to the far left are of representative wild-type sibling. No differences were observed among siblings of the different mutants. Scale bar = 10 μm. Mut, mutant; WT Sib, wild-type sibling.

To test what effects cilia gene mutations had on kinocilia morphology in hair cells, we stained mutants with acetylated tubulin. It has been shown previously that zebrafish *ift88* and *traf3ip* mutants show a loss of kinocilia (Tsujikawa and Malicki 2004; Omori *et al.* 2008). We confirmed these observations, and additionally saw a loss of kinocilia at 5 dpf in the retrograde IFT mutants *dync2h1* and *wdr35* (Figure 5A). Hair cells in *arl13b* mutants maintain their kinocilia at 5 dpf, although in some cases the kinocilia appear shortened as compared to wild-type siblings (Figure 5A). These observations are in agreement with previous work that has shown kidney ciliogenesis differs between IFT and *arl13b* mutants (Caspary *et al.* 2007; Duldulao *et al.* 2009). In contrast, all transition zone mutants we tested showed grossly normal kinocilia morphology (Figure 5B).

### Zebrafish cilia gene mutations show a delayed effect on hair cells

**Figure 6:**
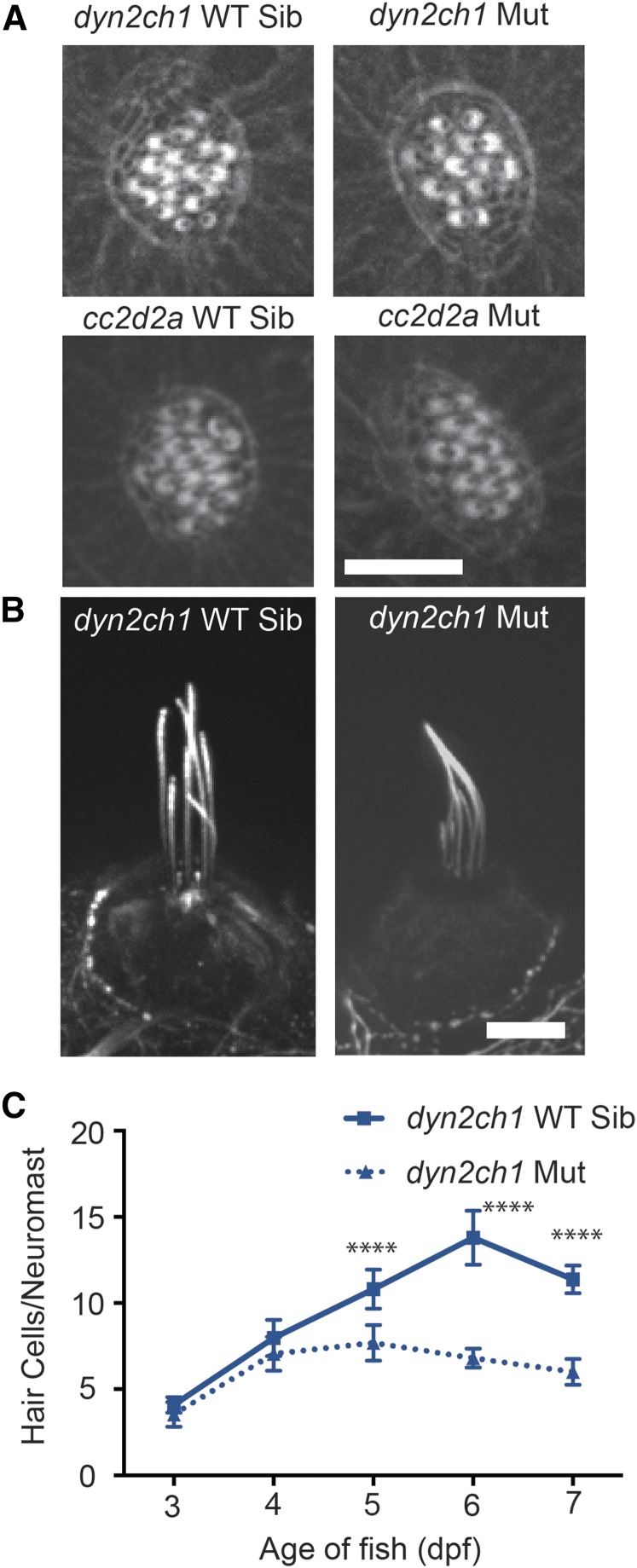
Early developmental defects are not seen in the hair cells of zebrafish cilia mutants. (A) Phalloidin labeling of the stereocilia of 5 dpf zebrafish hair cells show no gross morphological defects in *dync2h1* or *cc2d2a* mutants. Scale bar = 10 μm. (B) Earlier in development, at 3 dpf, *dync2h1* mutants have grossly normal kinocilia. Scale bar = 10 μm. (C) Decreases in hair cell number in the absence of neomycin in *dync2h1* mutants is not seen until 5 dpf. Data are shown as mean ± SD. *n* = 10 fish per group. **** *P* < 0.00001 by two-way ANOVA and Šídák multiple comparisons test. dpf, d post-fertilization; Mut, mutant; WT Sib, wild-type sibling.

Cilia-associated genes are believed to play a role in the development of stereocilia polarity in mammalian auditory hair cells (Ross *et al.* 2005; Jones *et al.* 2008), but not vestibular hair cells (Sipe and Lu 2011). Previous studies have also shown that stereocilia morphology of lateral line hair cells is grossly normal in zebrafish *ift88* mutants (Kindt *et al.* 2012). We examined stereocilia morphology in *dync2h1* and *cc2d2a* mutants using phalloidin labeling to visualize the actin-based stereocilia. In both cases we observed no obvious defects (Figure 6A), suggesting that gross defects in stereocilia formation are not the underlying cause for aminoglycoside-resistance in these mutants.

Defects in kinocilia morphology and inner ear hair cell number get worse with age in zebrafish *ift88* mutants (Tsujikawa and Malicki 2004). To test if this is also true for other cilia mutants, we examined *dync2h1* mutants at additional developmental time points. While kinocilia are never observed in 5 dpf *dync2h1* mutants, grossly normal appearing kinocilia were present in a subset of 3 dpf mutants (Figure 6B). Additionally, we found that at 3 and 4 dpf, unlike at latter ages, there was no significant decrease in control hair cell numbers between *dync2h1* mutants and wild-type siblings (Figure 6C). This delayed onset in defects could be due to maternal expression of cilia-associated genes, and may explain why stereocilia are able to develop normally. Maternal expression of cilia-associated genes is frequently observed in zebrafish (Huang and Schier 2009; Duldulao *et al.* 2009; Cao *et al.* 2010). We find that *dync2h1* mRNA is present in 2–4 cell zebrafish embryos, as assayed by RT-PCR (data not shown), supporting the notion that *dync2h1* is maternally expressed.

### IFT but not transition zone mutants show decreased mechanotransduction-dependent FM1-43 and neomycin loading

**Figure 7:**
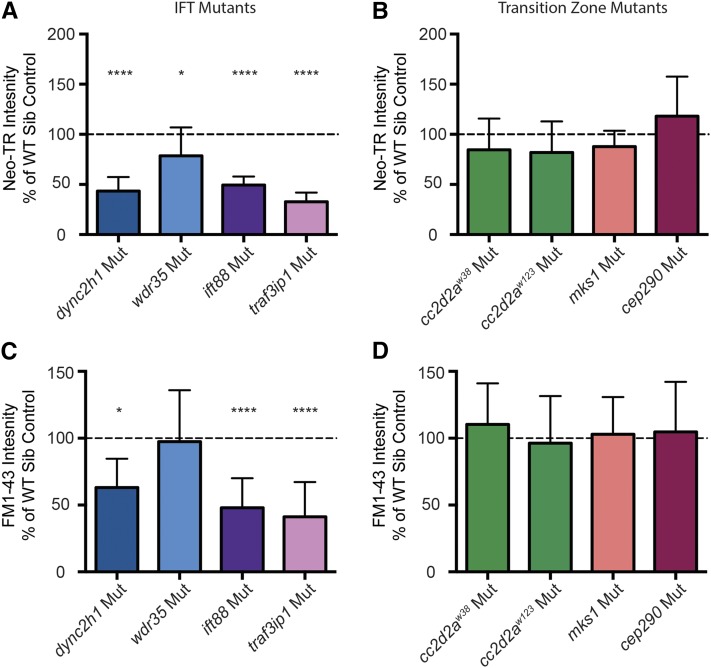
Intraflagellar transport (IFT) but not transition zone mutants show defects in neomycin and FM1-43 uptake. (A) Mutations in all four IFT genes cause a significant decrease in neomycin-Texas Red (neo-TR) loading. * *P* = 0.0152, **** *P* < 0.00001. For *dync2h1*, *ift88*, and *traf3ip*, *n* = 14–20 fish. For *wdr35*, *n* = 38 wild-type siblings and 10 mutants. (B) Mutations in the transition zone genes *cc2d2a (w38*, *P* = 0.1414, *w123*, *P* = 0.1191), *cep290* (*P* = 0.1414), and *mks1* (*P* = 0.0885) do not show a significant decrease in neo-TR loading. For *cc2d2a* and *cep290*, *n* = 14–21 fish. For *mks1*, *n* = 38 wild-type siblings and 14 mutants. (C) Mutations in *dync2h1*, *ift88*, and *traf3ip*, but not *wdr-35* (*P* = 0.8434), cause a significant decrease in rapid FM1-43 loading. * *P* = 0.0164, **** *P* < 0.00001. For *dync2h1*, *ift88*, and *traf3ip*, *n* = 14–20 fish. For *wdr35*, *n* = 37 wild-type siblings and 10 mutants. (D) Mutations in the transition zone genes *cc2d2a (w38*, *P* = 0.4273, *w123*, *P* = 0.7372), *cep290* (*P* = 0.6925), and *mks1* (*P* = 0.7851) do not show any decrease in FM1-43 loading. For *cc2d2a* and *cep290*, *n* = 12–18 fish. For *mks1*, *n* = 36 wild-type siblings and 12 mutants. Data are displayed as mean + SD, showing % of fluorescence intensity in the cell bodies of neuromast hair cells of mutants as compared to the average fluorescence intensity of wild-type siblings imaged at the same time. Statistics are calculated using Student’s or Welch’s *t*-test comparing mutants to wild-type siblings. Mut, mutant; WT Sib, wild-type sibling.

Previous mutants identified through genetic screening for resistance to neomycin-induced hair cell death have shown defects in hair cell loading of FM1-43 and aminoglycosides (Hailey *et al.* 2012; Stawicki *et al.* 2014). However, aminoglycoside loading appeared grossly normal in *cc2d2a^w38^* mutants (Owens *et al.* 2008). To test whether neomycin loading was altered in cilia mutants, we treated fish with neomycin that was covalently labeled with the fluorophore Texas Red (neomycin-TR), and quantified the fluorescent signal in the cell body of neuromast hair cells. We found significant decreases in neomycin-TR loading in all four IFT gene mutants tested, although the decrease was less dramatic in *wdr35* mutants than other mutants (Figure 7A). Mutations in *cc2d2a*, *mks1*, and *cep290* do not result in a significant decrease in neomycin-TR loading (Figure 7B).

The loading of aminoglycosides into hair cells requires mechanotransduction-activity (Gale *et al.* 2001; Marcotti *et al.* 2005; Alharazneh *et al.* 2011). To begin understanding why aminoglycoside uptake was diminished in IFT mutants, we examined the rapid uptake of the vital dye FM1-43. Previous work has shown that rapid uptake of FM-143 also requires mechanotransduction activity (Seiler and Nicolson 1999; Gale *et al.* 2001; Meyers *et al.* 2003). We found that mutations in both anterograde IFT genes *ift88* and *traf3ip*, as well as the retrograde IFT motor gene *dynch21*, lead to a significant decrease in FM1-43 loading; however, a mutation in the retrograde IFT adaptor gene *wdr35* had no effect on FM1-43 loading (Figure 7C). Mutations in the transition zone genes *cc2d2a*, *mks1*, and *cep290* also had no effect on FM1-43 loading (Figure 7D) as predicted from the neo-TR results (Figure 7B). These results show that while both IFT and transition zone genes play a role in aminoglycoside-induced hair cell death, their mechanisms of action in hair cells differ.

## Discussion

Cilia-associated genes have long been believed to primarily function in hair cells during development. Here, we have shown that multiple classes of cilia-associated genes play a role modulating the sensitivity of hair cells to aminoglycosides. Mutations in the anterograde IFT adaptor molecule genes *ift88* and *traf3ip*, as well as the gene for the retrograde IFT motor *dync2h1*, all lead to a decrease in control hair cell numbers, a strong resistance to neomycin-induced hair cell death, loss of kinocilia, and a decrease in hair cell loading of both FM1-43 and neomycin-TR, two processes known to be mechanotransduction-dependent. The later suggesting defects in mechanotransduction itself. A mutation in the retrograde IFT adaptor gene, *wdr35*, also caused a decrease in control hair cell numbers, a loss of kinocilia, and a comparable level of protection against neomycin-induced hair cell death as other IFT mutants. However, uptake of FM1-43 was not decreased in this mutant and the decrease in neomycin-TR loading was not as striking. There is precedence for *dync2h1* mutants showing different phenotypes than mutants in IFT-A complex genes (Ocbina *et al.* 2011; Liem *et al.* 2012), and this may be due to cargo differences between the different IFT-A adaptor molecules (Mukhopadhyay *et al.* 2010).

Mutations in the transition zone genes *cc2d2a*, *mks1*, and *cep290* lead to significant, but much more moderate, resistance in neomycin-induced hair cell death. These mutants did not have significant defects on control hair cell number, kinocilia morphology, or uptake of FM1-43 and neomycin-TR. Cc2d2a and Mks1 are believed to be in a complex at the cilia transition zone and to function similarly (Williams *et al.* 2011; Garcia-Gonzalo *et al.* 2011; Chih *et al.* 2012). In agreement with this observation, we see nearly identical levels of protection against neomycin-induced hair cell death in these two mutants, and a similar slight but insignificant reduction in neomycin-TR loading into hair cells. Cep290 has also been shown to interact with Cc2d2a (Gorden *et al.* 2008; Garcia-Gonzalo *et al.* 2011); however, it is believed to occupy a different region of the transition zone (Yang *et al.* 2015). The neomycin resistance we see in *cep290* mutants is not as strong as the resistance seen in *cc2d2a* or *mks1* mutants, suggesting that *cep290* functions slightly differently than the other two transition zone genes.

There have been conflicting reports on the role of transition zone genes in cilia formation. Cep290, Cc2d2a, and Mks1 have been implicated in ciliogenesis of cultured cells and a subset of ciliated tissues *in vivo* (Dawe *et al.* 2007; Tallila *et al.* 2008; Kim *et al.* 2008; Weatherbee *et al.* 2009; Garcia-Gonzalo *et al.* 2011). However, cilia formation has been shown to be unaffected in zebrafish injected with *cep290* antisense morpholino oligonucleotides (Sayer *et al.* 2006), *cc2d2a* zebrafish mutants (Bachmann-Gagescu *et al.* 2011), and the hair cells of *Mks1* mutant mice (Cui *et al.* 2011). Here we show that these genes are also not required for ciliogenesis in zebrafish hair cells. The tissue-specific role of transition zone genes in ciliogenesis highlights that this process is not uniform across cell types.

While we have shown novel roles for cilia-associated genes in hair cells through this work, a full understanding of the functions of these genes in hair cells remains to be elucidated. IFT genes *ift88*, *traf3ip*, and *dync2h1* appear to be important for hair cell mechanotransduction activity. We believe that their role in aminoglycoside-induced hair cell death is therefore through regulating the uptake of aminoglycosides into hair cells. While all three mutants lack kinocilia, we do not believe the kinocilia itself is responsible for this defect because *wdr35* mutants lack kinocilia but still show normal FM1-43 loading. A recent study has shown that IFT88 is important for the trafficking of Cadherin 23 and Harmonin (Blanco-Sánchez *et al.* 2014), molecules important for hair cell mechanotransduction activity (Di Palma *et al.* 2001; Söllner *et al.* 2004; Grillet *et al.* 2009). Hair cells contain microtubule tracks throughout their cytosol that extend into the actin-rich cuticular plate in the apical region of the cell (Jaeger *et al.* 1994). Therefore, in addition to anterograde IFT proteins’ role in kinociliary trafficking, they may move gene products important for mechanotransduction along microtubule tracks in the cytoplasm of hair cells to the apical region. As there is turnover of proteins at the tips of stereocilia (Zhang *et al.* 2012), and stereocilia tip link proteins Cadherin 23 and Protocadherin 15 are known to be rapidly replaced after damage (Zhao *et al.* 1996; Indzhykulian *et al.* 2013), retrograde IFT genes may also play a role in clearing protein from the apical region.

*wdr35* mutants show comparable protection to neomycin exposure as other IFT mutants, but no changes in hair cell loading of FM1-43, and a reduced effect on neomycin loading. These observations suggest that *wdr35* functions via a distinct mechanism in aminoglycoside-induced hair cell death compared to other IFT genes. Wdr35 has been shown to play a role in mitochondria cell death signaling in cultured cells (Fan *et al.* 2012), and mitochondria cell death signaling is believed to be involved in aminoglycoside-induced hair cell death (Cunningham *et al.* 2004; Matsui *et al.* 2004; Owens *et al.* 2007; Coffin *et al.* 2013). It is currently unclear how *wdr35* influences mitochondria cell death signaling; however, one possibility is through a role in mitochondria trafficking. IFT genes have been shown to be important for cytoplasmic microtubule morphology and dynamics (Cong *et al.* 2014; Bizet *et al.* 2015), and mitochondria are known to traffic along microtubules (Friede and Ho 1977; Frederick and Shaw 2007). An early step in aminoglycoside-induced cell death is the transfer of Ca^2+^ from the endoplasmic reticulum to the mitochondria (Esterberg *et al.* 2014). This step may be altered if mitochondria are mislocalized, preventing cell death from being initiated.

Finally, transition zone gene mutants appear to function distinctly from IFT mutants in aminoglycoside-induced hair cell death as they do not show as robust protection, and do not show a significant decrease in FM1-43 or aminoglycoside loading. *cep290* and *cc2d2a* have been shown to be important for protein trafficking (McEwen *et al.* 2007; Garcia-Gonzalo *et al.* 2011; Bachmann-Gagescu *et al.* 2015). While neomycin levels do not appear to be significantly reduced in hair cells of transition zone mutants, it is possible that the neomycin is being trafficked abnormally within the cell and that this modulates its toxicity. Further study into the mechanisms by which mutations in cilia-associated genes lead to protection against neomycin-induced hair cell death will help uncover the full role of cilia-associated genes in hair cells.

## Supplementary Material

Supplemental Material
