# Supplementary material for: Cilia-Associated Genes Play Differing Roles in Aminoglycoside-Induced Hair Cell Death in Zebrafish
Source: G3 (Bethesda). 2016 May 19;6(7):2225–35. doi: 10.1534/g3.116.030080 (PMC4938675; doi:10.1534/g3.116.030080)
Supplement: Supplemental Material [file supp_6_7_2225__index.html]

Cilia-Associated Genes Play Differing Roles in Aminoglycoside-Induced Hair Cell Death in Zebrafish — Supplemental Material 

# Cilia-Associated Genes Play Differing Roles in Aminoglycoside-Induced Hair Cell Death in Zebrafish

## Supplemental Material for Stawicki *et al.*, 2016

**Files in this Data Supplement:**

- Table S1 - Strains used in this study. (.pdf, 332 KB)
