# Supplementary material for: Cilia-Associated Genes Play Differing Roles in Aminoglycoside-Induced Hair Cell Death in Zebrafish
Source: G3 (Bethesda). 2016 May 19;6(7):2225–35. doi: 10.1534/g3.116.030080 (PMC4938675; doi:10.1534/g3.116.030080)
Supplement: Supplemental Material [file supp_g3.116.030080_TableS1.pdf]

Table S1 Strains used in this study

Gene	Allele	Nucleotide Change	Protein Change	How Generated	Reference
arl13b	*hi459Tg*	Proviral insertion in 5′-UTR	No protein product made	Proviral insertion	(Golling *et al.* 2002; Duldulao *et al.* 2009)
cc2d2a	*w38*	g1884a	W628X	ENU	(Owens *et al.* 2008)
cc2d2a	*w123*	t to g in 2nd residue of intron 20	Frame shift after R876 caused by splicing error	ENU	
cep290	*fh378*	Deletion of c294 & c295	Frame shift after A99	TALEN	
dync2h1	*w46*	g1452a	W484Stop	ENU	
ift88	*tz288*	t779a	L260Stop	ENU	(Brand *et al.* 1996; Tsujikawa and Malicki 2004)
mks1	*w152*	25 bp insertion after a181	Frame shift after S61	CRISPR	
traf3ip1	*tp49d*	c583t	R195Stop	ENU	(Brand *et al.* 1996; Omori *et al.* 2008)
wdr35	*w150*	t2426a	L809Stop	ENU	

Brand M., Heisenberg C. P., Warga R. M., Pelegri F., Karlstrom R. O., Beuchle D., Picker A., Jiang Y. J., Furutani-Seiki M., Eeden F. J. van, Granato M., Haffter P., Hammerschmidt M., Kane D. A., Kelsh R. N., Mullins M. C., Odenthal J., Nusslein-Volhard C., 1996 Mutations affecting development of the midline and general body shape during zebrafish embryogenesis. Development **123**: 129–142.

Duldulao N. A., Lee S., Sun Z., 2009 Cilia localization is essential for in vivo functions of the Joubert syndrome protein Arl13b/Scorpion. Development **136**: 4033–42.

Golling G., Amsterdam A., Sun Z., Antonelli M., Maldonado E., Chen W., Burgess S., Haldi M., Artzt K., Farrington S., Lin S.-Y., Nissen R. M., Hopkins N., 2002 Insertional mutagenesis in zebrafish rapidly identifies genes essential for early vertebrate development. Nat. Genet. **31**: 135–40.

Omori Y., Zhao C., Saras A., Mukhopadhyay S., Kim W., Furukawa T., Sengupta P., Veraksa A., Malicki J., 2008 Elipsa is an early determinant of ciliogenesis that links the IFT particle to membrane-associated small GTPase Rab8. Nat. Cell Biol. **10**: 437–44.

Owens K. N., Santos F., Roberts B., Linbo T., Coffin A. B., Knisely A. J., Simon J. A., Rubel E. W., Raible D. W., 2008 Identification of genetic and chemical modulators of zebrafish mechanosensory hair cell death. PLoS Genet. **4**: e1000020.

Tsujikawa M., Malicki J., 2004 Intraflagellar transport genes are essential for differentiation and

survival of vertebrate sensory neurons. Neuron **42**: 703–16.
